# Antimicrobial resistance surveillance in the AFHSC-GEIS network

**DOI:** 10.1186/1471-2458-11-S2-S8

**Published:** 2011-03-04

**Authors:** William G Meyer, Julie A Pavlin, Duane Hospenthal, Clinton K Murray, Kurt Jerke, Anthony Hawksworth, David Metzgar, Todd Myers, Douglas Walsh, Max Wu, Rosa Ergas, Uzo Chukwuma, Steven Tobias, John Klena, Isabelle Nakhla, Maha Talaat, Ryan Maves, Michael Ellis, Glenn Wortmann, David L Blazes, Luther Lindler

**Affiliations:** 1 Armed Forces Health Surveillance Center, 11800 Tech Rd, Silver Spring, MD 20904, USA; 2 Armed Forces Research Institute of Medical Sciences, 315/6 Rajavithi Road, Bangkok, Thailand 10400; 3 Brooke Army Medical Center, 3871 Roger Brooke Drive, Fort Sam Houston, TX 78234-6200, USA; 4 Landstuhl Regional Medical Center, Department of Immunology, CMR 402, Box 483, APO AE 09180, USA; 5 Naval Health Research Center, 140 Sylvester Road, San Diego, CA 92106, USA; 6 National Naval Medical Center, 8901 Wisconsin Avenue, Bethesda, MD 20889, USA; 7 U.S. Army Medical Research Unit-Kenya, U.S. Embassy, ATTN: MRU, United Nations Avenue, Post Office Box 606, Village Market, 00621 Nairobi, Kenya; 8 U.S. Navy and Marine Corps Public Health Center, 620 John Paul Jones Circle, Suite 1100, Portsmouth, VA 23708, USA; 9 U.S. Naval Medical Research Unit Number 2, Kompleks Pergudangan DEPKES R.I., JI. Percetakan Negara II Number 23, Jakarta, 10560, Indonesia; 10 U.S. Naval Medical Research Unit Number 3, Extension of Ramses Street, Adjacent to Abbassia Fever Hospital, Postal Code 11517, Cairo, Egypt; 11 U.S. Naval Medical Research Center Detachment, Centro Medico Naval “CMST,” Av. Venezuela CDRA 36, Callao 2, Lima, Peru; 12 Walter Reed Army Medical Center, 6900 Georgia Avenue Northwest, Washington, DC 20307, USA

## Abstract

International infectious disease surveillance has been conducted by the United States (U.S.) Department of Defense (DoD) for many years and has been consolidated within the Armed Forces Health Surveillance Center, Division of Global Emerging Infections Surveillance and Response System (AFHSC-GEIS) since 1998. This includes activities that monitor the presence of antimicrobial resistance among pathogens. AFHSC-GEIS partners work within DoD military treatment facilities and collaborate with host-nation civilian and military clinics, hospitals and university systems. The goals of these activities are to foster military force health protection and medical diplomacy. Surveillance activities include both community-acquired and health care-associated infections and have promoted the development of surveillance networks, centers of excellence and referral laboratories. Information technology applications have been utilized increasingly to aid in DoD-wide global surveillance for diseases significant to force health protection and global public health. This section documents the accomplishments and activities of the network through AFHSC-GEIS partners in 2009.

## Introduction and background

Antimicrobial resistance is a growing threat to the control of infectious disease globally and within the United States. Lethal organisms once thought to be on the decline are re-emerging with resistance to commonly used antimicrobials. Resistant organisms once acquired exclusively in hospital settings are now widely circulating in communities. Infections with resistant organisms not only result in greater severity and higher rates of morbidity and mortality, but also increase health care treatment costs and long-range expenses related to research and development of new drugs.

Since its inception, AFHSC-GEIS has a legacy of antimicrobial resistance surveillance. This activity continues as one of the five key focus areas for AFHSC-GEIS. Although few of the annual AFHSC-GEIS-funded proposals are singularly devoted to antimicrobial resistance, over several years the organization’s partners have developed diverse surveillance programs that often involve studies of microbial sensitivities to antibiotics. Many of these projects include outcomes that not only direct appropriate use of antimicrobials for individual patients and regional health planners, but are also aligned with DoD efforts of medical diplomacy with capacity building and investments in outbreak detection and response. These activities often involve collaborations with the host-nation civilian and military clinics and hospitals, as well as university systems. Other antimicrobial resistance-related programs represent collaborations within DoD for identification and tracking of infections at military medical facilities. Taken together, these activities are beneficial to both force health protection and the host nation in many interconnected levels. The following is a synopsis of selected partner activities from 2009.

## 2009 contributions

### Pathogens in Southeast Asia

Over the last several years, a dramatic rise in antibiotic resistance of enteric pathogens, including enterotoxigenic *Escherichia coli* (ETEC), *Shigella*, *Salmonella* and *Campylobacter* has been documented in Southeast and South Asia [1]. Many hospitals and laboratories in this region lacked the essential infrastructure and capability to isolate and identify enteric pathogens and to reliably test antimicrobial resistance. Several U.S. Naval Medical Research Unit Number 2 (NAMRU-2) projects involved laboratory-based microbiologic surveillance of patients presenting for the care of febrile illnesses in host-nation civilian and military clinics and hospitals scattered around the regions. These efforts not only help determine the etiology of febrile illnesses and antimicrobial sensitivity patterns in these areas but also provide essential host-nation laboratory capacity building and training.

In an effort to determine the epidemiology and etiologies of acute febrile illness of unknown origin among persons seeking medical care in Cambodia, NAMRU-2 established a five-year hospital-based surveillance study commencing in December 2006. Depending on the patients’ presenting complaints, nasal/throat swabs, serum samples, blood cultures, malaria smears and stool samples were obtained from 4,751 patients with fever at as many as nine different health care centers over the years. Although the most prevalent pathogens isolated among Cambodian patients were influenza, dengue and malaria, the studies also identified diseases such as leptospirosis, hantavirus and hepatitis A, B and E, as well as diseases caused by rickettsial infections. Bacterial organisms collected from these studies were analyzed for antimicrobial resistance. The surveillance also identified the first cases of multidrug-resistant *Salmonella typhi* with reduced susceptibility to fluoroquinolones in Cambodia [2].

Another NAMRU-2 effort, the Indonesian Pediatric Diarrhea Surveillance program, links six distinct geographic sites on five islands of the Indonesian archipelago. The ongoing multi-year collaborative effort has collected more than 12,000 specimens from patients with acute diarrhea symptoms. Bacterial pathogens were identified in 1,142 cases (11 percent), with *Campylobacter* and *Shigella* species being the most prevalent etiologies isolated. *Vibrio cholerae* was the most common *Vibrio* species identified. Shigellosis was identified as the cause of diarrhea in 300 (2 percent) of the cases. Antimicrobial susceptibility testing of *Shigella* samples demonstrated very high levels of resistance to trimethoprim-sulfamethoxazole, often used as the first-line antibiotic to treat children with diarrhea in Indonesia [3].

*Campylobacter* species were identified in 314 cases of children presenting with diarrhea to participating hospitals and health clinics in Jakarta, Makassar and Mataram. Antimicrobial susceptibility testing to *C. jejuni* identified rates of ciprofloxacin resistance as high as 65 percent with several cases exhibiting macrolide resistance. Similar patterns were observed for isolates of *C. coli*. An increase in minimum inhibitory concentrations (MIC) for several antibiotics, including ciprofloxacin, was found when analyzing results over the time span of specimen collections. NAMRU-2 implemented a real-time polymerase chain reaction (PCR) assay to discriminate between *Campylobacter* isolate wild-type and mutant alleles that can confer resistance to fluoroquinolones. The study found the majority of resistant isolates possessed this mutation. Several strains that were negative by PCR but resistant by MIC testing are being further characterized by sequence analysis of the quinolone resistance-determining region in an attempt to identify other novel mutations that may confer resistance [3].

### Cholera surveillance

Investigators at the U.S. Armed Forces Research Institute of Medical Sciences (AFRIMS) responded to a large outbreak of severe diarrhea that affected over 70,000 people in 2009. They found 52 percent of the 158 samples were positive for *Vibrio cholera* strain O1, 18 percent were heat-labile toxin-expressing ETEC and 13 percent were heat-stable toxin-expressing ETEC. All *V. cholerae* strains isolated from the outbreak were resistant to nalidixic acid and trimethoprim-sulfamethoxazole, but they were sensitive to tetracycline, ciprofloxacin, norfloxacin and ampicillin [4].

The *V. cholerae* and rotavirus reference center for the Middle East and Africa established by the U.S. Naval Medical Research Unit Number 3 (NAMRU-3) performed antibiotic sensitivity testing of 303 archived *V. cholerae* samples. The center’s studies demonstrate widespread resistance to streptomycin, trimethoprim-sulfamethoxazole and nalidixic acid. Resistance to ampicillin and chloramphenicol was observed only in isolates from Somalia, and resistance to tetracycline was limited to single isolates from Qatar and Somalia [5].

### Health care-associated pathogens

Health care-associated infections and antimicrobial resistance have emerged as important public health problems in both developed and resource-poor countries, as well as among DoD personnel. Increased surveillance in hospitals in developing countries may determine risk factors (e.g., overuse of antibiotics, counterfeit drugs or deficiencies in local patient management guidelines and policies) that are different from those in developed countries. This may also define high-risk medical practices and enable the hospitals to tailor and implement intervention plans to reduce infection rates in resource-limited settings. These surveillance activities may become examples that other developing countries can follow in their attempts to reduce nosocomial infection rates and increase the safety of health care systems in regions where U.S. personnel and travelers visit frequently. Furthermore, knowledge about the genetic determinants of antimicrobial resistance can be very important for tailoring antibiotic policies and tracing the nosocomial spread of pathogens. Molecular characterization studies are essential tools that may help reduce nosocomial infection mortality rates and the cost of treating antibiotic-resistant infections, and limit inappropriate antibiotic use.

More than 30,000 U.S. military personnel have been wounded in action while serving in Iraq or Afghanistan [6], and many of these patients are at risk for serious complications. Wound infections have been a common complication of these injuries and, as in previous wars, are often caused by gram-negative organisms. Minimal information is available about the mechanism of antimicrobial resistance of bacterial infections in Middle Eastern countries. Working with various regional host-nation partners, the laboratories have been able to document the geographic spread of antimicrobial resistance in common organisms. This vital information is used to effectively advise local and national health care leaders about necessary changes to antimicrobial formularies and provides important information on appropriate antibiotic treatment for troops deployed in the Middle East [7]. Extended spectrum beta-lactamase-producing gram-negative rods and *Acinetobacter baumannii* are major causes of infections in health care settings. Many of these nosocomial infections are difficult to treat with antibiotics, and the antibiotic-resistant organisms that cause them are increasingly being seen in community-acquired infections [8].

In Egypt and Jordan, NAMRU-3 has been engaged in surveillance of health care-acquired infections and antimicrobial resistance with emphasis on intensive care units [9]. Researchers analyzed 124 highly resistant gram-negative rods that were collected as part of this surveillance network. Isolates were characterized phenotypically by standard bacteriological procedures and antimicrobial susceptibility profiles were obtained using Clinical and Laboratory Standards Institute (CLSI) criteria. Major mechanisms of antimicrobial resistance in isolates confirmed as either extended-spectrum beta-lactamase-producing bacteria or *Acinetobacter baumannii* were further characterized by pulsed-field gel electrophoresis (PFGE), plasmid profiling, PCR amplification and DNA sequencing.

The resulting antimicrobial resistance profile classified 46 (37 percent) of the isolates as multidrug-resistant organisms. Nineteen isolates (15 percent) were resistant to imipenem and 20 (16 percent) were resistant to meropenem. Sixteen isolates (13 percent) were resistant to both carbapenems. All isolates were sensitive to colistin and were also sensitive or intermediately sensitive to minocycline. Antimicrobial resistance testing using the Etest^®^ strip method indicated production of metallo-beta-lactamase in 18 (14 percent) of isolates.

The NAMRU-3 surveillance of health care-acquired infections and antimicrobial resistance demonstrated that gram-negative organisms constituted the majority of isolates in both countries (61 percent in Egypt and 65.2 percent in Jordan) [8]. In Jordan, *Klebsiella* species were the most commonly isolated gram-negative pathogens (17.4 percent of all isolates), whereas in Egypt, *Klebsiella* species and *A. baumannii* were equally prevalent among the gram-negative bacilli with each organism accounting for 15.4 percent of the isolates. Not surprisingly, high rates of antimicrobial resistance were reported in hospitals in both countries. Extended-spectrum beta-lactamase producer rates among *E. coli* isolates were 64.3 and 66.7 percent in Egypt and Jordan, respectively. *Klebsiella* species showed extended-spectrum beta-lactamase producer rates of 57.1 percent in Egypt and 75 percent in Jordan. In Egypt, resistance of *Pseudomonas aeruginosa* to imipenem was 58.8 percent, and almost 88 percent of *Staphylococcus aureus* isolates were resistant to methicillin. The higher rates of antimicrobial resistance in specific intensive-care units (ICUs) encourage the establishment of better-informed antimicrobial use policies and implementation of stricter infection control practices. These mitigations help decrease antimicrobial resistance rates and reduce the risk of spread to the community.

### *Acinetobacter* infections

Antimicrobial-resistant strains of bacteria threaten U.S. military personnel deployed in the Middle East and Afghanistan from combat- and non-combat-related infections caused by these highly resistant pathogens [10]. *Acinetobacter baumannii-calcoaceticus* complex, *P. aeruginosa*, *Klebsiella* and *E. coli* are common pathogens, but, compared to past wars, the acquisition of multidrug-resistant isolates appears to be significantly increased [11]. *A. baumannii* is a common nosocomial challenge in Egypt and has emerged as one of the important opportunistic pathogens in hospitalized patients throughout the world. Additionally, these infections plague DoD and Veterans Affairs medical treatment facilities and contribute to prolonged hospital stays. Outbreaks of *Acinetobacter* infections are becoming increasingly common among patients in ICUs, surgical units and burn units [12].

The severe impact of an *Acinetobacter* outbreak on hospital operations requires a quick assessment of the potential spread of these infections. Comparison of *A. baumannii* PFGE patterns from Egypt with isolates collected at military treatment facilities in the U.S. showed high levels of genetic variability among collections. The majority of the *Acinetobacter* isolates cultured from hospitalized injured personnel have been multidrug-resistant, limiting the use of some empiric antibiotics for treatment of wound infections [11].

Continuous surveillance with genetic characterization of *Acinetobacter* is the ideal method to direct infection-control measures or altering of antimicrobial regimens. Molecular genotyping of these isolates enhances infection control of personnel wounded in the Middle East and the possible nosocomial transmission of these organisms within the DoD hospital system. Characterization of the multi-drug resistant organisms via PFGE genotyping will help identify possible sources of infection and lead to strategies for employing appropriate antibiotics and isolation practices.

Landstuhl Regional Medical Center (LRMC) has an active surveillance protocol for *A. baumannii*. All incoming patients from Operation Iraqi Freedom, Operation Enduring Freedom and Africa are screened for colonization of *Acinetobacter*. Between October 2008 and March 2009, more than 500 *A. baumannii* isolates were processed. Three main clusters of 90 percent similarity were identified and account for 66 percent of the strains examined. This comparison of *Acinetobacter* genotypes significantly benefits infection control efforts by helping identify potential sources of spread, particularly those that occur during the evacuation chain from the battlefield to U.S.-based hospitals. This genotyping data has been used for infectious disease surveillance and infection control at LRMC and thereby improved total patient care.

In January 2009, an increase in the number of *A. baumannii* infections with similar antibiograms in the LRMC ICU was observed [13]. The three strains were shown to be genotypically similar. While the actual source was not determined, the infection control team adopted additional measures, and further spread did not occur. The data from the *Acinetobacter* studies, including the PFGE, antibiotic susceptibility and plasmid profiling, will provide valuable information regarding the epidemiology and evolution of *A. baumannii* from the onset of Operation Iraqi Freedom.

Collection of this data allows researchers to identify strains of interest for further detailed molecular work (e.g., genome sequencing). The PFGE data collected at LRMC is shared with other military treatment facilities. The DiversiLab and PFGE data generated at LRMC allow AFHSC-GEIS and DoD to make more informed funding decisions by comparing the two systems in terms of cost, ease of use and quality of data.

A unique three-year collaborative effort championed by Dr. Luther Lindler, the GEIS Acinetobacter Surveillance Initiative, established standard operating procedures for PFGE of *Acinetobacter* species among laboratories at Brooke Army Medical Center (BAMC), Walter Reed Army Institute of Research (WRAIR), Walter Reed Army Medical Center and LRMC. Some of the samples processed were sent to BAMC and WRAIR for comparison with the samples of all participating military treatment facilities. Some samples processed by each of the partner laboratories were sent to BAMC and WRAIR for analysis, generating a systematic collection of PFGE patterns-identified strains infecting DoD personnel. This enhanced the capabilities of DoD to determine the initial geographic location of contamination as well as the spread and prevention of virulent strains of gram-negative organisms originating from wound infections. Specific isolate information coupled with patient outcomes led to the identification of specific virulence factors within particularly virulent strains and guides the use of specific or novel antibiotic treatments [14].

### Drug resistance studies at Brook Army Medical Center

The primary objective of the Center of Excellence for Leptospirosis at BAMC is developing reliable molecular diagnostic techniques based on PCR for the disease. The center has also assessed the antimicrobial therapies for leptospirosis using *in vitro* and *in vivo* models. After developing a calorimetric system to test antimicrobial resistance by an *in vitro* model to various leptospiral strains from around the world, the center has continued to collect novel strains from around the world to test while assessing a broad array of serovars against older and newer antimicrobial agents.

Antimicrobial agents that have been tested to date include older agents such as amikacin, cefazolin, ceftazidime, cephalexin, colistin, fosfomycin, gentamicin, metronidazole, minocycline, polymyxin, rifampin, sulfamethoxazole, tobramycin, trimethoprim, imipenem, and vancomycin [15-18]. Newer and novel agents including tigecycline, doripenem, cethromycin, CEM-101 and CEM-102 also have been tested. In addition, the more active *in vitro* antimicrobial agents have been characterized further in a lethal hamster model for *in vivo* activity. This work has enabled the center to respond to specific requests by clinicians in the developing world to determine if older more commonly used antimicrobials not used in the U.S., such as chloramphenicol, have activity.

Additionally, BAMC has maintained a referral laboratory at the San Antonio Military Medical Center (Texas) to support DoD outbreak investigations and other epidemiological investigations and research. This center provides centralized molecular biology support to characterize multidrug-resistant bacteria pathogens and has been involved in multiple studies. BAMC developed a collaborative research relationship with Fort Sill (Oklahoma) to support a methicillin-resistant *Staphylococcus aureus* (MRSA) colonization study supported through AFHSC-GEIS and an Infectious Disease Clinical Research Program-sponsored project of chlorhexidine-impregnated cloths to prevent skin and soft tissue infections in U.S. Marine officer candidates. Additionally, they performed resistance and virulence factor gene analysis for *Klebsiella pneumonia* and developed and analyzed numerous multidrug-resistant pathogens and possible outbreaks including analysis of MRSA isolates recovered from the U.S. Army Institute of Surgical Research burn unit during the past 25 years [19,20].

Further studies at BAMC evaluated the resistance mechanisms of *Enterobacteriaceae* as well as extended-spectrum beta-lactamase-producing *Klebsiella* isolates and their impact within a burn unit. They also studied the efficacy of topical agents for *Klebsiella*, *Pseudomonas*, MRSA, and *Acinetobacter baumannii-calcoaceticus* complex in the burn unit [21,22]. These projects included the study of multidrug-resistant *Klebsiella*, *Acinetobacter baumannii-calcoaceticus*, and *Pseudomonas* infections over time within a patient and between patients. A major focus of the BAMC group is to further characterize antimicrobial activity against *Acinetobacter baumannii-calcoaceticus* complex [23].

Clinical laboratory testing methods and broth microdilution were used to define the susceptibility phenotypes of 107 single-patient isolates from blood and wound infections to 15 antimicrobial agents. Genetic relationships were determined by PFGE, and isolates were screened for selected resistance determinants. The isolates were resistant to an average of nine agents and four antimicrobial classes, with 92 percent meeting a definition of multidrug-resistance.

The most active agents were colistin (MIC_90_ 0.5 µg/mL, 99 percent susceptible) and minocycline (MIC_90_ 4 µg/mL, 90 percent susceptible). Carbapenems, traditionally reserved for multidrug-resistant infections, were relatively inactive (imipenem MIC_90_ 0.5 µg/mL, 38 percent S). Fifty-two percent of isolates carried the OXA-23 carbapenemase, which substantially degraded the activity of imipenem (78.4 percent S without, versus 1.8 percent S with OXA-23 present). Rifampin has promising *in vitro* activity (MIC_90_ 4 µg/mL); however, no susceptibility breakpoints have been defined. Aminoglycosides also had very limited activity (amikacin MIC_90_ ≥ 256 µg/mL, 16.8 precent S; gentamicin MIC_90_ ≥32 µg/mL, 4.7 percent S; tobramycin MIC_90_ ≥ 32 µg/mL, 27.1 percent S). Aminoglycoside-modifying enzymes were heterogeneous in these isolates, and poorly predictive of the aminoglycoside susceptibility phenotype.

Nearly half (49.5 percent) of the isolates carried the class 1 integron, a marker for the acquisition of cassettes containing multiple antimicrobial resistance genes. Of 107 isolates, 106 (99 percent) carried at least one resistance determinant. Significant inaccuracies were found in some clinical testing methods for tetracyclines, aminoglycosides and, to a lesser extent, carbapenems. Identifying the most prevalent resistance mechanisms, optimal susceptibility testing and judicious use of antimicrobial agents may help preserve the last remaining agents with activity against multidrug-resistant bacteria.

Overall these projects have enabled BAMC to leverage its various programs to collaborate with internal and external partners to improve treatment of combat-related injury infections and multi-drug resistant infections by assessing local delivery of antibiotics in animal models and characterizing novel resistant bacteria. It has also enabled better characterization of multi-drug-resistant infection rates throughout the military health care system from point of injury through tertiary care referral hospitals in the U.S.

### Antimicrobial resistance in military trainee populations

Another ongoing AFHSC-GEIS-supported activity at Naval Health Research Center (NHRC), San Diego, characterizes the clinical isolates of *Streptococcus pyogenes* from U.S. military basic trainees [24]. Group A *S. pyogenes* (GAS) infections are common in young adults and may present clinically as pharyngitis, scarlet fever or invasive disease. GAS is also associated with post-infectious sequelae, including rheumatic heart disease and glomerulonephritis. Acute GAS infections remain susceptible to penicillin but resistance to macrolide antibiotics has been noted in recent years.

Antibiotics are frequently used for prophylaxis of recruits against infections; therefore characterization of GAS isolates is necessary in these populations. Ongoing surveillance since 1998 has demonstrated continued susceptibility to penicillin and low-level resistance to macrolides and other antibiotics in GAS isolates collected at nine recruit training sites.

Macrolide resistance is of particular concern because this class of antibiotics is often used for prophylaxis and treatment of individuals who are allergic to penicillin. NHRC tested 2,837 GAS isolates from recruits since the study’s inception in 1998. Among 240 isolates collected in 2009, in comparison with previous annual studies, lower resistance was seen with erythromycin (6.6 percent), while higher resistance was seen for tetracycline (7.0 percent), clindamycin (4.3 percent), and levofloxacin (6.5 percent). Higher levofloxacin resistance was seen in 2009 at Marine Corps Recruit Depot, Parris Island (South Carolina) and higher clindamycin, erythromycin and tetracycline resistance was seen at Fort Benning (Georgia). Additionally, M protein gene (*emm*) typing of *S. pyogenes* performed by NHRC has demonstrated associations of certain *emm* types to resistance and virulence. Other *emm* types have been shown to be more likely associated with outbreaks among U.S. military trainees.

Monitoring high-risk populations such as recruits for emergence of potentially virulent strains could lead to early interventions that may prevent outbreaks and reduce morbidity. The most common *emm* gene types among trainees were 3, 5, 44, 6 and 75. *Emm* type 75 was associated with increased levels of erythromycin resistance, but without apparent increased virulence. *Emm* type 5 was less common but is one of the potentially more virulent strains and was implicated in several outbreaks in 2006 and 2007. A high degree of correlation exists in the temporal distribution of strain patterns between multiple sites suggesting a concerted strain turnover pattern occurring on a larger scale.

These results demonstrated the sensitivity and specificity of a recently developed, rapid, high-throughput strain identification technology and provided the basis for a method of rapid inferential prediction of clinically relevant characteristics using rapid strain typing methods. This surveillance also provided information on circulating strains for GAS vaccine development initiatives.

### Electronic surveillance of antimicrobial resistance

A completely different approach using electronic data sources for antimicrobial resistance surveillance was undertaken by the Navy Marine Corps Public Health Center (NMCPHC). NMCPHC developed algorithms and tools to interpret Health Level 7 (HL7) data derived from the DoD Composite Health Care System for surveillance of diseases significant to public health [25]. Use of this data for the surveillance of antibiotic resistance feeds data into BacLink and WHONET, tools developed by the World Health Organization (WHO). Using its experience with inpatient and outpatient encounter records, laboratory and pharmacy data, and other medical and personnel databases, NMCPHC explored trends in disease burden and antibiotic-resistant microorganisms.

The DoD-wide HL7 electronic microbiology laboratory data were restructured to rapidly identify and monitor emerging antimicrobial resistance in organisms, such as *A.baumannii*, *K. pneumoniae*, *P. aeruginosa* and other pathogens of public health concern. The HL7 data stream provides an opportunity for active surveillance of trends in antibiotic resistance and near-real-time response to significant health threats, especially in high-risk populations.

These capabilities were used to enhance surveillance and understanding of trends in emerging pathogens and antibiotic resistance as well as to answer requests for information about invasive MRSA and *S. pneumoniae* in the Military Health System beneficiary population and describe skin and soft tissue infections among DoD members. More than 175,000 skin and soft tissue cases were identified among DoD active-duty military service member medical encounters between October 2006 and May 2008.

Seventy-four percent of cultured skin and soft tissue infection cases were associated with *S. aureus*, followed by coagulase-negative *Staphylococcus* (6 percent), *E. coli* (2 percent), *P. aeruginosa* (1 percent) and *Proteus mirabilis* (1 percent). More than half of the *S. aureus* isolates tested for oxacillin sensitivity were defined as MRSA. MRSA isolates were not only resistant to oxacillin but also to erythromycin (90 percent). Methicillin-sensitive *S. aureus* (MSSA) isolates were sensitive to other commonly-used antibiotics, including trimethoprim-sulfamethoxazole and vancomycin. HL7 outpatient pharmacy data showed that the antibiotics used to treat both types of infection were very similar; trimethoprim-sulfamethoxazole was prescribed frequently for both MRSA (67 percent) and MSSA (52 percent).

The first iteration of an NMCPHC antimicrobial-resistant organism surveillance website displays antibiograms and high-profile organism counts, as well as similar data broken out by service and region [26]. The project includes the use of WHONET to generate facility-specific, DoD-wide and regional antibiograms for comparison and assessment of trends external to their own patient populations. The methodology used to restructure antimicrobial data was validated by comparing the sensitivity profiles prepared by military treatment facilities to the NMCPHC sensitivity profiles from the restructured data. The validation process is ongoing.

The importance of electronic surveillance is demonstrated further in the NMCPHC analysis of the study, *Acinetobacter* species Infections: Trends in Active-Duty Servicemembers. The investigation identified more than 6,300 *Acinetobacter* isolates found in 2,467 DoD active-duty servicemembers between 2005 and 2008. *Acinetobacter* species isolates from wound specimens made up 34 percent of active-duty servicemember isolates (n=2,138) and showed levels of susceptibility between 45 percent and 80 percent to all of the commonly prescribed antibiotics reviewed throughout the study time period; 95 percent of these isolates were *Acinetobacter baumannii-calcoaceticus* complex [25].

Isolates identified from blood specimens made up 6.5 percent of all active-duty service member isolates (n=409). Overall susceptibility of these isolates to amikacin was 36 percent, imipenem susceptibility was 57 percent and colistin susceptibility was 33 percent. Meropenem results for these isolates were quite limited, with only 15 isolates tested and an overall susceptibility of 7 percent [25].

A similar electronic study of upper respiratory infections and antibiotic resistance among U.S. Navy recruits with upper respiratory infection-associated medical encounters and microbiology records document isolation of *Streptococcus* in 92 percent and *P. aeruginosa* in 3 percent of 1,022 laboratory specimens [25].

## Future direction and initiatives

Since the inception of DoD-GEIS and now AFHSC-GEIS, multiple partners have studied various aspects of antimicrobial resistance with a multitude of methods. This section documents the 2009 partner accomplishments and activities.

The increasing prevalence of emerging antimicrobial-resistant infections remains one of the greatest threats to global health and will continue to be a major concern for AFHSC-GEIS. To successfully counter this threat, the global network will need to be united and coordinated. The three-year GEIS Acinetobacter Surveillance Initiative demonstrated the value of standardized operating procedures and central specimen archives in expanding the knowledge base for these unique, but all too common, infections. AFHSC-GEIS-funded partners will need to develop closer collaborations to best understand the various components involved with global antibiotic-resistant organism surveillance. AFHSC-GEIS has begun initiatives such as subject matter expert steering committees to better direct and coordinate funded proposals as a means of identifying surveillance gaps, avoiding redundancies and assuring state-of-the art technologies.

The creation of an Antimicrobial-Resistant Organism Steering Committee is planned for fiscal year 2011 and will help develop a unified global surveillance plan to combat this common enemy. Increased and sustained surveillance capabilities that can rapidly identify genetic and phenotypic patterns of resistance are essential tools for surveillance in the ever-changing field of microbiology and antimicrobial resistance.

The ability to track antimicrobial resistance in organisms causing disease in DoD beneficiaries is essential for infectious disease and public health leaders to formulate policy and determine appropriate actions for mitigation. AFHSC-GEIS will continue to fund studies that increase its knowledge of the forces and mechanisms that create resistant organisms so that the battle against antimicrobial resistance can be waged more effectively.

## Conclusion

Infectious diseases have always been a major threat to U.S. military forces and global public health. Antimicrobial resistance surveillance has been a pillar of military force health protection and global public health for AFHSC-GEIS since its creation in 1998. AFHSC-GEIS funding has enhanced the ability of partner laboratories to maintain robust infectious disease surveillance.

A significant spin-off of these efforts is the acquisition of multiple isolates of microorganisms infecting patients from various regions of the world which can be further analyzed for antimicrobial resistance. The rate and spread of antimicrobial resistance can be tracked and helps to direct patient care, antibiotic prescribing practices and national policy.

To better understand the global picture of infectious disease, it is essential to use standardized nomenclature and laboratory procedures in order to correctly identify the causative microorganisms. Additionally, the various reports must be collated if the data is to be relevant to DoD populations spread around the world. AFHSC-GEIS-funded partners have continued to contribute greatly to essential research and development of techniques that further the investigation of disease causing organisms. Rapidly changing technologies and computer applications speed the process of analysis of the organisms and allow more educated and informed policies and interventions to better treat and prevent infectious disease threats. This report has provided a synopsis of recent antimicrobial resistance surveillance accomplishments achieved by AFHSC-GEIS partners.

## Competing interests

The authors declare that they have no competing interests.
